# Donor’s age influences outcome in haploidentical hematopoietic stem cell transplantation with post-transplant cyclophosphamide - a single center experience

**DOI:** 10.1007/s00277-024-05848-z

**Published:** 2024-06-15

**Authors:** Patrycja Zielińska, Agata Wieczorkiewicz-Kabut, Krzysztof Białas, Anna Koclęga, Karolina Gruenpeter, Anna Kopińska, Krzysztof Woźniczka, Izabela Noster, Tomasz Gromek, Jarosław Czyż, Sebastian Grosicki, Agnieszka Wierzbowska, Jacek Krzanowski, Aleksandra Butrym, Grzegorz Helbig

**Affiliations:** 1 Department of Hematology and Bone Marrow Transplantation, Medical University of Silesia, 40-032 Katowice, Dąbrowski Street 25, Katowice, Poland; 2 Department of Hematooncology and Bone Marrow Transplantation, Medical Univeristy of Lublin, Lublin, Poland; 3 Department of Hematology, Faculty of Medicine, Collegium Medicum in Bydgoszcz, Nicolaus Copernicus University, Bydgoszcz, Poland; 4 Department of Hematology and Cancer Prevention, Chorzów, Faculty of Public Health in Bytom, Medical University in Katowice, Chorzów, Poland; 5 Department of Hematology, Medical University of Lodz, Łódź, Poland; 6 Brzozow Oncology Center, Brzozów, Poland; 7 Department of Cancer Prevention and Therapy, Wroclaw Medical University, Wałbrzych, Poland

**Keywords:** Acute leukemia, Donor, Haploidentical stem cell transplantation, Infections, Outcome, Posttransplant cyclophosphamide

## Abstract

Haploidentical stem cell transplantation (haplo-SCT) using post-transplantation cyclophosphamide (post-Cy) is considered a reasonable therapeutic option for patients who lack matched donor or who urgently need transplant procedure due to high risk disease. We analyzed the results of haplo-SCT performed in years 2018–2023. Eighty one patients (46 males) at median age of 52 years underwent haplo-SCT using peripheral blood as a stem cell source in most cases. Indications included hematological malignancies (acute leukemias in 88% of cases). In 25 cases (31%) transplantation was performed in relapsed/refractory disease. Majority of patients (61%) presented with very high and high disease risk index (DRI). Conditioning regimens were as follows: nonmyeloablative − 46 cases (57%), myeloablative – in 18 (22%) and reduced intensity – 17(20%). 90% of patients engrafted. All patients received unified immunosuppressive treatment (post-Cy/TAC/MMF). Median follow-up time was 12 months The cumulative incidence of acute and chronic GVHD was 37.5% and 37.6%, respectively. Estimated 2-year overall survival (OS) was 43.1% and donor’s age was the only factor influencing survival. The 2-year progression-free survival (PFS) was 42.5%, whereas relapse incidence (RI) − 35%. The cumulative incidence of non-relapse mortality (NRM) was 44% and was mostly due to infections. Haplo-SCT is a feasible treatment option for hematological patients. Younger donor improves post-transplant survival. Strategies to reduce infection-related mortality and relapse rate remain a challenge.

## Introduction

Haploidentical hematopoietic stem cell transplantation (haplo-SCT) with post-transplant cyclophosphamide (post-Cy) to control alloreactivity has revolutionized the field of allogeneic stem cell transplantation. The probability of identifying an HLA-matched sibling or unrelated donor presents some limitations which makes a haploidentical donor of considerable importance [1]. Haploidentical family – related donor not only shortens the time to transplantation due to immediate availability but also enables post-transplant donor-derived cellular therapies which aim to prevent disease relapse [2].

Nowadays, new possibilities emerged for haploidentical family donor transplantation. Due to introduction of post-Cy strategy, the outcome of matched/mismatched unrelated SCT and haploidentical ones are largely comparable [3, 4] even when performed in active disease [5]. Moreover, the results of sibling transplantations are similar to haplo-SCT as well [6–8].

Cyclophosphamide is administered after haplo-SCT and inhibits both graft rejection and graft versus host (GVH) effect while allowing proper engraftment [9]. Cyclophosphamide induced tolerance selectively targets alloreactive T cells while preserving immunity to infection [9]. These arguments make a haploidentical transplantation a feasible option. SCT from a related donor mismatched for one HLA haplotype is considered as an effective strategy for patients with high risk hematological neoplasms, especially those who lack HLA matched donor for whom prolonged donor search could be disadvantageous [10].

## Patients and methods

The retrospective analysis included adult patients diagnosed with hematological malignancies in our institutional database of medical records who underwent haplo-SCT between March 2018 and March 2023. Disease risk index (DRI) in our group of patients was calculated according to previously published criteria [11]. All patients had a donor who was at least 5/10 HLA identical with the patient. Donor-specific antibodies (DSAs) were tested in all patients. DSA titers with mean fluorescence intensity (MFI) below 1000 were considered as clinically not important [12]. In other cases patients were qualified for desensitization procedures with therapeutic plasma exchange (TPA) and/or immunoglobulin 1 g/kg (IVIG) infusion and/or single dose rituximab (375 mg/m2). The Hematopoietic Cell Transplant Co-morbidity Index (HCT-CI) was calculated for each patient according to Sorror et al. [13]. The conditioning regimens varied depending on the type of the disease, remission status and patient’s comorbidities. Stem cell source was initially unmanipulated bone marrow, but in majority of patients – G-CSF stimulated peripheral blood. Graft-versus-host disease (GVHD) prophylaxis consisted of post-transplant cyclophosphamide (50 mg/kg/day) on days 3 and 4 after transplantation with mesna followed by tacrolimus and mycophenolate mofetil starting from day 5.

On admission to transplant unit urine culture as well as throat and anal swab cultures were performed to assess possible colonization with resistant bacterial strains. All patients received antimicrobial prophylaxis with posaconazole and acyclovir in peritransplant period. Patients also received standard *Pneumocystis jiroveci* prophylaxis with co-trimoxazole at least 6 months after the transplantation or longer in patients with prolonged immunosuppressive treatment. Letermovir as a prophylaxis of cytomegalovirus (CMV) reactivation was used in a very limited group of patients due to lack of availability of that drug in Poland at that time.

In case of neutropenic fever the antibiotics were escalated either empirically or – in case of previously identified pathogens - targeted antimicrobial therapy was given. In cases with markedly elevated infectious markers and /or clinical deterioration, despite broad spectrum antibiotics used, serum galactomannan and mannan tests were performed and antifungal treatment was escalated. Blood monitoring for CMV reactivation using quantitative PCR (polymerase chain reaction) was performed weekly for the first month and then at least once bi-weekly up to 100 days after transplantation. In our center a viral load of 800 copies/ml was established as an optimal cut off for initiating preemptive therapy with valganciclovir. The treatment was continued for 2 weeks till CMV eradication. BK virus assessment by qualitative PCR was performed in patients with dysuria and/or hematuria. All patients with confirmed BK infection were given hyperhydration and bladder irrigation via three-way catheter. Intravesical cidofovir was given weekly in refractory cases with high urine viral load. For patients transplanted in active disease, our policy was based on early, but careful tapering of immunosuppressive therapy starting from day 30 after transplantation with cautious monitoring of donor chimerism. Irradiated leukodepleted blood products transfusions and other supportive care measures were per institution practices.

Definitions of engraftment and graft failure were consistent with previously published criteria [14]. Day 30 donor chimerism in unsorted bone marrow cells was assessed after the transplantation using STR (short tandem repeats) method to confirm donor-origin hemopoiesis. Acute and chronic graft versus-host-disease were diagnosed and graded according to standard criteria [14]. Due to retrospective nature of the study some data were incomplete or not available.

### Statistical analysis

Pre-transplant patient characteristics were expressed as the median and interquartile range (IQR) for continuous and frequencies and proportions for categorical variables. The quantitative variables were characterized by the arithmetic mean of standard deviation or median or max/min (range) and 95% confidence interval. The qualitative variables were presented with the use of count and percentage. Estimates for progression free survival (PFS) and overall survival (OS) probabilities were determined through Kaplan-Meier calculations. Non-relapse mortality (NRM) and relapse incidence (RI) were estimated using cumulative incidence curves in a competing risk setting, death in remission being treated as a competing event for relapse. To estimate acute or chronic GVHD, relapse and death were considered as competing events. Univariate analyses were performed using the log-rank test for OS. Variables considered were: type of conditioning, CMV sero-status between donor and the recipient, donor/recipient sex combination, HCT-CI index, DRI score and AB0 match. Multivariate analyses were performed using the Cox proportional hazards regression model. Results were expressed as the hazard ratio (HR) with 95% confidence interval (95% CI. The statistical significance level was fixed at *p* = 0.05. The statistical calculations were performed using TIBCO Software Inc. (2017) Statistica (data analysis software system), version 13 (http://statistica.io).

## Results

### Patient characteristics

Between March 2018 and March 2023 eighty one patients underwent haplo-SCT. The median age at SCT was 52 years (IQR 40–64) and included 46 males (57%) and 35 females (43%). Indications for SCT included the following hematological malignancies: acute myeloid leukemia in 56 cases (69%), acute lymphoblastic leukemia – 15 cases (19%), non-Hodgkin lymphoma – 4 (5%), multiple myeloma – 2 (3%), primary myelofibrosis – 2 (3%), chronic myeloid leukemia – 1 (1%) and Hodgkin lymphoma 1 (1%). In 12 patients (15%) haplo-SCT was their second stem cell transplantation performed due to relapse or progression of the underlying disease. Six of them (7%) had a history of previous autologous procedure, 3 (4%) – previous sibling transplantation and 4 (5%) – unrelated transplantation. Majority of patients (52%) presented with high DRI, 32 patients (39%) – intermediate and 7 (9%) – very high. Twenty-five patients (31%) were transplanted due to relapsed/refractory disease.

The majority of patients (83%) had good performance status at transplant: 0–1 according to the Eastern Cooperative Oncology Group (ECOG) score. Only 13 patients presented ECOG 2 (16%), and 1 patient was scored 3 (1%). Nonetheless, nearly half of patients had a history of chronic disease or serious infection prior to transplant procedure. HCT-CI was calculated as low in 27 (34%), intermediate in 44 patients (54%), and high in 10 patients (12%).

Descriptive characteristics of patients are summarized in Table 1.

**Table 1:** Patient characteristics

Patients, *n*, %	81 (100)
Age in years, median, IQR	52 (40–64)
Sex (male/female), n, %	46 (57)/35 (43)
Diagnosis: AML/ALL/other n, %	56(69)/15(19)/10 (13)
Previous SCT: autologous/sibling/unrelated, n, %	6 (7)/3 (4)/4 (5)
DRI, n, %: very high/high/intermediate/low, n, %	7 (9)/42(52)/32(39)/0(0)
Relapsed/refractory disease, n, %	25 (31%)
HCT-CI, n, %: low/intermediate/high	27 (34)/ 44 (54)/ 10 (12)

*ALL*: acute lymphoblastic leukemia; *AML*: acute myeloid leukemia; *DRI*: disease risk index; *HCT-CI*: hematopoietic cell transplantation-specific comorbidity index; *IQR*: interquartile range; *SCT*; stem cell transplantation

### Transplant data

Median donor age was 38 years (IQR 32–44), and 63% (51) of donors were male. Eligible haploidentical donors included related family members who shared at least one HLA haplotype. Donors were first-degree relatives or siblings who were HLA-haploidentical based on molecular typing at HLA-A, -B, -C, -DRB1, and -DQB1. Haploidentical family donor was an offspring in most cases – 51 (63%), sibling – 22 (27%), parent − 8 (9%) and uncle in 1 case only (1%). In 22 cases (27%) HLA match was higher than 5/10. In 6 cases only the patients underwent desensitization. All of them eliminated DSA to acceptable values.

57 patients (71%) presented with unfavorable CMV serological status in donor-recipient (D/R) pairs before transplant procedure (cases D+/R-, cases D+/R+). In one case only (1%) the status was favorable (D-/R-), and in the remaining 23 cases (28%) the status was intermediate (D-/R+) in terms of CMV reactivation risk after transplantation. Forty-seven patients (58%) presented with AB0 group matched donors. The major AB0 group mismatch was detected in 13 cases (16%), in 20 (25%) – minor, and in 1 (1%) donor-recipient pair major and minor AB0 group incompatibility (bidirectional mismatch) was present.

In 46 patients (57%) nonmyeloablative conditioning (NMA) based on standard Baltimore protocol containing fludarabine (30 mg/m2 IV, renally adjusted), Cy (14.5 mg/kg IV), total body irradiation (200 cGy) was administered, whereas in 18 cases (22%) myeloablative conditioning (MAC) regimen was used. In the remaining 17 cases (20%) reduced intensity protocols were used. Stem cell source was as follows: G-CSF (granulocyte-colony stimulating factor) mobilized peripheral blood in 97% (79 cases), bone marrow in 3% (2 cases). In our study group median transplanted CD34^+^ cell count was 5.35 of ×10^6^/kg recipient b.w. (IQR 4.37–6.34), whereas median CD3 + cell count was 16.98 × 10^7^/kg recipient b.w (IQR 12.3-21.65).

All patients received posttransplant cyclophosphamide as stated above. The immunosuppressive treatment was unified in all patients and contained mycophenolate mofetil (MMF) at dose 15 mg/kg body weight and tacrolimus (TAC) starting from day 5 (with dose adjusted to maintain a level of 5–15 ng/mL). Engraftment was achieved in 90% of patients and presented as follows: for neutrophils − 21 days (IQR:19–23); for platelets − 19 days (IQR: 14–24).

The transplant data are presented in Table 2.

**Table 2:** Transplant data

Patients, *n*, %	81 (100%)
Donor age in years, median, IQR	38 (32–44)
Female donor to male recipient, n, %	16 (20)
CMV status donor-recipient (+/- or +/+), n, %	57 (71)
ABO matched grafts, n, %	47 (58)
Stem cell source: bone marrow/peripheral blood; n, %	2 (3)/ 79 (97)
Relationship of haploidentical family donor: child/ sibling/ parent/ uncle	51(63)/ 22(27)/8(9)/ 1(1)
Conditioning regimen, n, %	
myeloablative (TT-BU-FLU/ FLU-TBI 1200 cGy) nonmyeloablative (FLU-CY-TBI 200 cGy) reduced intensity	11 (14)/ 7 (9) 46 (57) 17 (20)
Number of transplanted CD34^+^cells x10^6^/kg, median, IQR	5.35 (4.37–6.34)
Number of transplanted CD3^+^ cells x10^7^/kg, median, IQR	16.98 (12.3-21.65)
Days to neutrophil engraftment, median, IQR Days to platelet engraftment, median, IQR	21 (19–23)
	19 (14–24)
Day 30 donor chimerism in %, median, IQR	99.25 (98.5–100)

*BU*: busulfan; *CMV*: cytomegalovirus; *CY*: cyclophosphamide; *FLU*: fludarabine; *IQR*: interquartile range; *TBI*: total body irradiation; *TT*: thiotepa

### Survival outcome

Median follow-up was 12 months (IQR 4–20). Data on day + 30 donor chimerism was available for all but 2 patients (98%). Median percentage of donor chimerism was 99.25% (IQR 98.5–100). The 2-year probability of overall survival (OS) was 43.1% (95% CI: 30.5–55.6).

The main cause of death was relapse of the primary disease, which was observed in 19 cases (24%). The 2-year cumulative incidence of relapse incidence (RI) was 35.0% (95% CI: 20.5–59.6), whereas the 2-year progression-free survival (PFS) was 42.5% (95% CI: 30.1–54.9). The second most frequent cause of fatal outcome was infection, which occurred in 15 patients (19%). In general, 2-year non-relapse mortality (NRM) was 44.0% (95% CI: 28.0-69.1). Sixty-three patients (78%) developed infectious complications within 30 days after transplantation, but in 2 cases only it resulted in death before recovery of hematopoietic system. Overall, 51 patients (63%) were diagnosed with bacterial bloodstream infections (BSI) caused by: *Staphylococcus epidermidis* (5), *Klebsiella pneumoniae* (4), *Escherichia coli* (3), *Pseudomonas aeruginosa* (3) *Staphylococcus hemolyticus* (2), *Enterococcus faecalis* (1) and fungal bloodstream growth was confirmed in one case (Candida albicans). Serious infections included: pneumonia in 21 cases - *Klebsiella pneumoniae ESBL* (1), *Enterococcus faecium* (1), *Aspergillus spp.* (7), *Candida spp*. (7), *Pneumocystis jiroveci* (7), infection in the site of central vein catheter (1), enteritis due to *Clostridioides difficile* infection (4). Viral complications included: hemorrhagic cystitis caused by BKV (11), SARS-Cov2 infection (9), and CMV reactivation (15). COVID19 resulted in fatal pneumonia in 3 patients.

The other causes of post-transplant mortality were as follows: steroid-refractory GVHD in 3 patients (4%), others – 1 case (1%). The data was missing in 2 cases (3%).

The incidence of primary graft failure was 4% (3 patients). Six patients were re-transplanted, 3 of them due to primary graft failure, the remaining 3 patients - due to relapse of the primary disease. Of note is that two out of these six patients are still alive. Secondary graft failure among patients with initial engraftment was not observed.

**Fig. 1:**
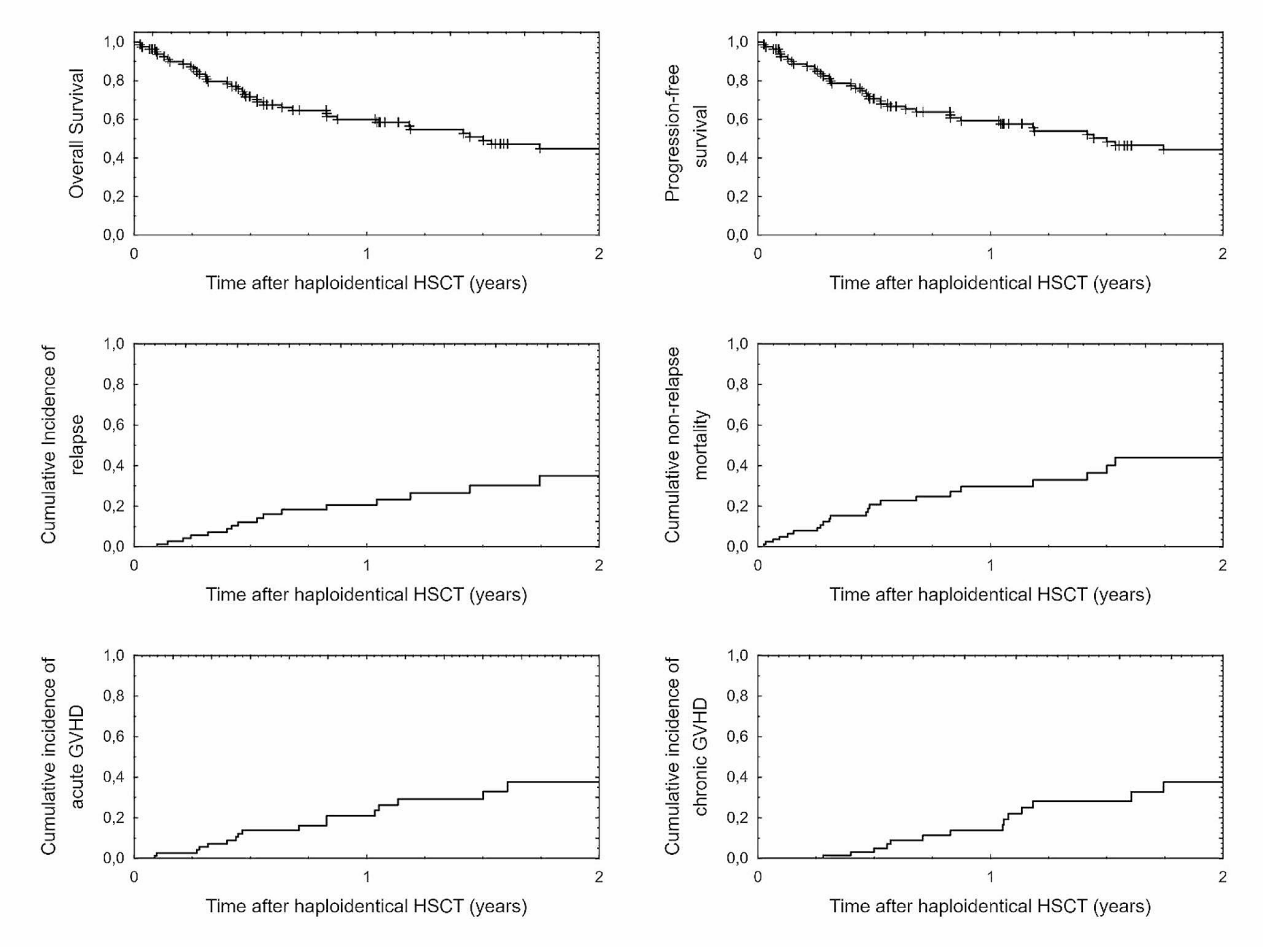
Two-year overall survival, progression-free survival, relapse incidence and non-relapse mortality and cumulative incidence of acute and chronic GVHD in patients who underwent haploidentical transplantation

Twenty six patients developed acute GVHD symptoms, including 5 patients with grade III-IV. Chronic GVHD was observed in 18 patients, but only 2 of them presented with extensive chronic GVHD. The 2-year cumulative incidence of acute and chronic GVHD was 37.5% (95% CI: 22.5–62.5) and 37.6% (95% CI:21.6–65.4), respectively. In general, 41 patients (51%) were alive at last contact. All transplant outcome data are presented in Table 3; Fig. 1.

**Table 3:** Transplant outcome

Patients; *n*, %	81 (100)
Primary graft failure, n, %	3 (4)
Acute GVHD II-IV, n,%	26 (32)
Chronic GVHD, n, %	18 (22)
Cause of death, n, %	
relapse infections GVHD other unknown	19 (24) 15 (19) 3 (4) 1 (1) 2 (3)
Alive at last contact, n, %	41 (51)
Follow-up time in months, median, IQR	12 (4–20)

*GVHD*: graft-versus-host disease; *IQR*: interquartile range

Multivariate analysis showed that haploidentical transplantations from older donors are associated with significantly lower OS (*p* = 0.03). Other analyzed parameters were not statistically significant. See Table 4 for details.

**Table 4:** Univariate and multivariate analysis for OS

	Univariate		Multivariate	
	HR (95%CI)	*P*-value	HR (95%CI)	*P*-value
patient age	1.00 (0.98–1.02)	0.9307	1.00 (0.97–1.04)	0.8947
donor age	1.03 (1.00-1.05)	0.0525	1.04 (1.00-1.07)	**0.0316**
female donor/ male recipient	1.47 (0.70–3.10)	0.3128	0.61 (0.26–1.40)	0.2437
other combinations	0.68 (0.32–1.44)	0.3128	1.64 (0.71–3.78)	0.2437
non-MAC conditioning	1.12 (0.56–2.26)	0.7464	1.00 (0.26–3.81)	0.9985
MAC conditioning	0.89 (0.44–1.79)	0.7464	1.00 (0.26–3.82)	0.9985
CMV status donor/recipient				
+/+	1.01 (0.53–1.93)	0.9835	1.16 (0.11–12.04)	0.9037
-/+	0.97 (0.48–1.95)	0.9334	0.93 (0.08-11.0)	0.9525
+/-	1.00 (0.24–4.16)	0.9985	1.10 (0.05–23.64)	0.9527
-/-	1.18 (0.16–8.59)	0.8714	0.87 (0.08–9.02)	0.9037
HCT-CI				
low	0.81 (0.13–5.09)	0.7871	0.71 (0.09–6.21)	0.6892
intermediate	1.51 (0.19–12.06)	08437	1.29 (0.15–11.24)	0.7194
high	2.09 (0.23–18.81)	0.3873	1.36 (0.13–14.27)	0.6998
DRI				
intermediate	1.36 (0.71–2.62)	0.3519	1.56 (0.70–3.47)	0.2761
high	0.86 (0.46–1.60)	0.6341	0.64 (0.29–1.43)	0.2761
very high	0.69 (0.27–1.77)	0.4390	0.71 (0.19–2.61)	0.6067
AB0 mismatch				
none	0.87 (0.47–1.63)	0.6745	1.21 (0.15–9.77)	0.8604
minor	1.37 (0.71–2.63)	0.3501	0.81 (0.12–5.65)	0.8359
major	0.85 (0.36–2.04)	0.7244	1.62 (0.28–9.37)	0.5878

*CMV*: cytomegalovirus; *DRI*: disease risk index; *HCT-CI*: hematopoietic cell transplantation-specific comorbidity index; *MAC*: myeloablative conditioning; *non-MAC*: reduced intensity and nonmyeloablative conditioning

## Discussion

Allogeneic stem cell transplantation is the only potentially curative treatment for many patients with hematologic malignancies. However, one of the major obstacles so far has been identification of a fully-matched donor. The selection of a donor has focused on minimizing alloreactivity between the immune systems of the donor and recipient, thus minimizing graft-versus-host disease and graft rejection which translated into better overall outcome. In general, the intensity of alloreactivity is proportional to the degree of HLA mismatch between donor and recipient [15]. Currently available evidence did not prove that the degree of donor-recipient HLA disparity in the setting of haplo-SCT with post-Cy has a significant impact on survival [16–18]. The majority of haploidentical donors are first-degree related family members such as parents, children or siblings. In general, if a suitable first-degree related donor is not available, non-first-degree related donors may remain a rational option [19]. The analysis of outcome of haploidentical transplantations in AML patients did not reveal any significant differences in OS, NRM, RI and GVHD risk between first-degree related haplo-donors and non-first degree ones [19]. An EBMT study comprised a total of 645 AML patients who received a haploidentical graft from a donor with either 2–3 of 8 HLA antigen mismatches (*n* = 180) or with 4 of 8 HLA antigen mismatches (*n* = 465). They found no difference in terms of GVHD occurrence, 2-year OS, LFS, RI and NRM, indicating adverse cytogenetics as the most important factor of poor survival [20]. Therefore, the degree of HLA-mismatch should not be considered as an important factor in choosing the best haploidentical donor.

The most widely used protocol includes use of high-dose cyclophosphamide in post-transplant setting which decreases alloreactive T cells but preserves non-alloreactive T cells, thus not affecting the engraftment rates [21]. So far collected data recommend to choose firstly offsprings, then male haplo-donor (sibling, father) over mother [22]. There was no difference between haploidentical sibling and offspring donor in terms of graft failure [23]. In our study group, there was prevalence of offsprings (63%) as haplo-donors, with only 30 female donors (37%). Female donors are regarded as potentially more immunogenic donors when compared to males especially when transplanted to male recipients. It was suspected that female donors in haplo setting may present higher HLA disparity and as a consequence provide higher GVHD risk [18, 24]. However, our data did not find female donor to male recipient transplantation as more hazardous in terms of survival. Probably the only donor factor in haplo setting which seems to be crucial is donor age (below or equal to 40 years is considered to be beneficial) [25]. It was stated that younger donors are less likely to present clonal hematopoiesis. Younger donors are also considered to have better total nucleated cell and CD34 + cell yield. Nonetheless, some data indicating donor age as an important risk factor affecting patient survival after haploidentical transplantation are conflicting [26]. Median donor age in our study group was 38. We found that younger donor age was associated with better survival.

In the study comparing HLA-matched HSCT with haploidentical bone marrow transplantation with post-Cy, the authors used the Disease Risk Index to present risk-stratified outcomes. They found that 3-year overall survival rates were 70% and 73% in low-risk disease, 47% and 49% in intermediate-risk disease, and 25% and 37% in high/very high-risk disease in HLA-matched and haplo-HSCT, respectively [27]. However, we did not find any significant correlations between DRI score and the transplant outcome in our study group.

We also found no impact of AB0 incompatibility on post-transplant survival as this finding was in line with other studies [28, 29].

As it was mentioned above, post-Cy functions by selective impairment of alloreactive T cells, while sparing suppressive regulatory T cells. Such selective T-cell modulation is stated as a probable factor that effects cellular immunity directed against CMV [30]. Recent data indicate that post-Cy contributes significantly to the development of CMV infection, regardless of donor source, especially in seropositive recipients [30]. Cytomegalovirus reactivation prophylaxis with letermovir was proved to reduce CMV infection incidence and all-cause mortality [31]. Nowadays, all post-Cy seropositive recipients or those with a seropositive donor should be regarded as high risk for CMV infection, and prophylaxis should be strongly considered [31]. In Poland letermovir has been reimbursed and widely used in seropositive patients receiving allogeneic stem cell transplantation since July 2022. It provides an explanation why only 7 out of 77 eligible (seropositive) patients in our study group underwent prophylactic treatment with letermovir. We found 14 (18%) CMV reactivations until day 100 post transplantation (none of them occurred in letermovir group). Nonetheless, CMV serostatus was not found to be a relevant risk factor in our study group (see Table 4 for details).

Despite several advances that have significantly improved the survival rate after allogeneic SCT, severe infections, especially bloodstream infections (BSI) remain a challenge and a major cause of non-relapse mortality [32, 33]. Cyclophosphamide causes mucosal damage which may be in part responsible for increased infections rate and may predispose to bacteremia especially in patients previously colonized with multidrug resistant strains [32]. In a large Spanish study encompassing 235 adult haploidentical transplantation recipients the overall incidence of bloodstream infection by gram-positive and gram-negative bacteria at 37 months was 51% and 46%, respectively [34]. The incidence of viral infections (in pre-letermovir era) was 69%, while Epstein Barr Virus infections occurred in 10% of patients and hemorrhagic cystitis in 35% of cases, whereas invasive fungal infections occurred in 11% [34]. The authors found that severe infections were the most common causes of NRM after haplo-SCT using post-Cy. The data are similar to our findings. In our study group infection-related deaths accounted for 19% (15 cases) of fatal outcomes, which was the higher rate when considering NRM factors. In another French study infections accounted for 45.7% of haplo-SCT recipients [33]. In our study group 78% of patients developed at least one infection in a 30-day posttransplant period, including 51 BSI (63%), 35 viral infections (43%) and 28 invasive fungal infections (34%).

## Conclusions

HLA-haploidentical hematopoietic stem cell transplantation is now one of the most commonly used alternative donor technique using high-dose posttransplant cyclophosphamide. The choice of the best haplo-donor remains a challenge, but younger donors should be favored. The most important question is how to lower the relapse rate as relapse of the underlying disease is the primary driver of mortality in patients treated with this transplant platform. The other challenge that needs to be addressed in the nearest future is to lower the infection rate as it is also an important factor influencing the survival in haploidentical transplant recipients.

## Data Availability

No datasets were generated or analysed during the current study.
